# Untargeted Lipidomics of Non-Small Cell Lung Carcinoma Demonstrates Differentially Abundant Lipid Classes in Cancer vs. Non-Cancer Tissue

**DOI:** 10.3390/metabo11110740

**Published:** 2021-10-28

**Authors:** Joshua M. Mitchell, Robert M. Flight, Hunter N. B. Moseley

**Affiliations:** 1 Department of Molecular & Cellular Biochemistry, University of Kentucky, Lexington, KY 40536, USA; jmmitchell@lanl.gov; 2 Markey Cancer Center, University of Kentucky, Lexington, KY 40536, USA; robert.flight@uky.edu; 3 Resource Center for Stable Isotope Resolved Metabolomics, University of Kentucky, Lexington, KY 40536, USA; 4 Institute for Biomedical Informatics, University of Kentucky, Lexington, KY 40536, USA; 5 Department of Toxicology and Cancer Biology, University of Kentucky, Lexington, KY 40536, USA

**Keywords:** non-small cell lung carcinoma, lipidomics, Fourier-transform mass spectrometry, SMIRFE

## Abstract

Lung cancer remains the leading cause of cancer death worldwide and non-small cell lung carcinoma (NSCLC) represents 85% of newly diagnosed lung cancers. In this study, we utilized our untargeted assignment tool Small Molecule Isotope Resolved Formula Enumerator (SMIRFE) and ultra-high-resolution Fourier transform mass spectrometry to examine lipid profile differences between paired cancerous and non-cancerous lung tissue samples from 86 patients with suspected stage I or IIA primary NSCLC. Correlation and co-occurrence analysis revealed significant lipid profile differences between cancer and non-cancer samples. Further analysis of machine-learned lipid categories for the differentially abundant molecular formulas identified a high abundance sterol, high abundance and high m/z sphingolipid, and low abundance glycerophospholipid metabolic phenotype across the NSCLC samples. At the class level, higher abundances of sterol esters and lower abundances of cardiolipins were observed suggesting altered stearoyl-CoA desaturase 1 (SCD1) or acetyl-CoA acetyltransferase (ACAT1) activity and altered human cardiolipin synthase 1 or lysocardiolipin acyltransferase activity respectively, the latter of which is known to confer apoptotic resistance. The presence of a shared metabolic phenotype across a variety of genetically distinct NSCLC subtypes suggests that this phenotype is necessary for NSCLC development and may result from multiple distinct genetic lesions. Thus, targeting the shared affected pathways may be beneficial for a variety of genetically distinct NSCLC subtypes.

## 1. Introduction

Lung cancer remains the most common cause of cancer death worldwide [1] with approximately 85% of newly diagnosed lung cancers belonging to the non-small cell lung carcinoma subtype (NSCLC) [2]. The high mortality of lung cancer, NSCLC included, is partially explained by the insidious and silent nature of the progression of early-stage disease, but also the lack of effective therapeutic options for advanced disease. Although improvements have been made in the fields of NSCLC treatment, especially for adenocarcinomas with actionable mutations such as epidermal growth factor receptor (EGFR), anaplastic lymphoma kinase (ALK), certain Kirsten rat sarcoma virus (K-RAS) variants, and more recently with checkpoint inhibitors [3], resistance often sets in [4,5,6]; and the overall 5-year survival rate remains low (<30%) [7].

Treatment options for NSCLC vary with disease stage as well as a genetic subtype. For low-stage disease, surgery remains the most common and most effective treatment option [8], especially when combined with adjuvant chemotherapeutic drugs [9]. For advanced disease, chemotherapy and/or radiotherapy [10,11,12,13] are the primary first-line treatment option. Therapeutics targeting the epidermal growth factor receptor (EGFR) [14,15], which is often mutated in NSCLC tumors in patients of East-Asian origin [16] and female never smokers, vascular endothelial growth factor (VEGF) [17,18,19,20], anaplastic lymphoma kinase (ALK), and the programmed death protein 1 and programmed death ligand-protein 1 (PD-1/PD-L1) immune checkpoint [21] have increased the number of therapeutic options available for patients with advanced disease. However, while offering different side-effect profiles than traditional chemotherapy [22], overall survival of advanced NSCLC remains poor [23] and many patients fail to express these particular biomarkers and are unsuitable for these therapies.

Finding drug targets for every genetic subtype of NSCLC would be very challenging and time-consuming. Alternatively, it is well known that cancer metabolism differs substantially from non-cancer metabolism due to metabolic reprogramming, a phenomenon that is essential to cancer development [24]. As multiple genetic mutations may result in the same metabolic phenotypes, drugs targeting these metabolic processes may be efficacious for multiple genetic subtypes of NSCLC.

There are many examples of metabolic reprogramming in NSCLC [25,26,27]. In particular, stable isotope labeling studies in vivo have shown that both glycolysis and the tricarboxylic acid (TCA) cycle are highly active in many NSCLC tumors, resulting in higher rates of glucose oxidation in NSCLC compared to surrounding non-NSCLC tissue [25]. Increased TCA activity provides the precursors needed for several anabolic processes needed for cell proliferation, but this requires concomitant increased anapleurosis, such as enhanced pyruvate carboxylation [27], to maintain TCA cycling. Additionally, many NSCLC tumors oxidize several other substrates with a preference for non-glucose substrates such as lactate [28] and glutamine [29,30,31] from the tumor microenvironment, especially at higher perfusion rates [25].

Lipid metabolism is also commonly altered in NSCLC. Key enzymes for lipid metabolism such as ATP citrate lyase (ACLY) [32], fatty acid synthase (FASN) [33], and stearoyl-CoA desaturase 1 (SCD1) [34] can be differentially expressed in NSCLC compared to non-cancer tissue. Overexpression of these genes enables enhanced production of many lipid classes and is also correlated with poorer clinical outcomes and tumor aggressiveness [34,35,36,37,38]. Additionally, previous studies have demonstrated an association between serum cholesterol and survival in patients with resectable NSCLC [39] (as well as other forms of cancer [40,41]) and an association between membrane cholesterol content and EGFR signaling activity [42]. Collectively, these findings suggest that altered lipid production plays a key role in the development and progression of NSCLC and that enzymes in these pathways are promising drug targets.

An alternative to the costly de novo development of novel therapeutics is the repurposing of existing pharmaceuticals for new indications. For example, previous observational studies have suggested that patients prescribed statins, inhibitors of 3-hydroxy-3-methyl-glutaryl-CoA reductase in the mevalonate pathway, have better overall survival rates for a variety of cancers including NSCLC [43], even at late stages [44], and in patients undergoing EGFR inhibitor therapy [45]. The difference in survival benefit between NSCLC stages and treatments combined with the observation that randomized controlled trials have had limited success replicating the survival benefit [45] seen in retrospective studies suggests that only some subtypes of NSCLC may be statin responsive. If patients with statin-responsive molecular subtypes of NSCLC can be identified, statins could play a supporting role in the treatment of NSCLC. Additionally, it remains unclear how statins result in a survival benefit mechanistically. Statins have multiple known off-target effects besides inhibition of sterol biosynthesis including anti-inflammation [46], immunomodulation [47], and angiogenesis inhibition [48], all of which may contribute to this possible survival benefit. However, if this effect could be attributed to inhibition of endogenous sterol production, other inhibitors of the mevalonate pathway such as nitrogenous bisphosphonates [49] could have a role in the treatment of NSCLC.

As these examples illustrate, an improved understanding of the metabolic differences between NSCLC and non-cancerous lung tissue represents a major first step in constructing more complete models of NSCLC progression and ultimately the development of more effective therapeutics. Advances in ultra-high resolution mass spectrometry, particularly Fourier-transform mass spectrometry (FT-MS), provide significant analytical improvements, including the ability to resolve distinct isotopologues, and the detection of lower abundance metabolites. These capabilities combined with our in-house data processing pipeline, artifact mitigation [50], and untargeted assignment method called Small Molecule Isotope Resolved Formula Enumeration (SMIRFE) [51,52] enables the assignment of molecular formulas to spectral features observed in NSCLC-derived lipid extracts without bias due to the incompleteness of existing metabolic databases [53,54]. These assignments can be classified into lipid category and class using our machine learning methods [55] to investigate changes that occur in lipid profiles at the lipid category level.

## 2. Results

### 2.1. Mass Spectrometry Data Processing, Assignment Ambiguity and Quality Control of Samples

Ambiguous assignments and high data sparsity are largely unavoidable due to the exponential growth of molecular formula search space at higher m/z [51], biological and analytical variance between biological units, sample preparation, and limitations in dynamic range. Elemental molecular formula (EMF) voting identified consistently assigned (i.e., corresponded) peaks and filtered these features to those present in at least 25% of samples for a given sample class to reduce data sparsity which in turn allows for the meaningful use of more traditional statistical approaches such as principal component analysis (PCA) that do not handle high data sparsity well. Even so, the high data sparsity present in the dataset significantly contributes to the low percent variance observed in the top principal components.

Additionally, our quality control measures allow us to detect outlier spectra that could represent failed sample preparation, poor injection, or non-primary or non-NSCLC tumors. Initially, 179 patient samples were included in the analysis. After the removal of 22 samples from unrelated secondary metastatic tumors and benign granulomatous tissue and of 12 outlier samples based on quality control, 145 samples remained for differential abundance analysis.

### 2.2. PCA and Sample Correlation Heatmap Shows Separation of Cancer and Non-Cancer Samples

**Figure 1:**
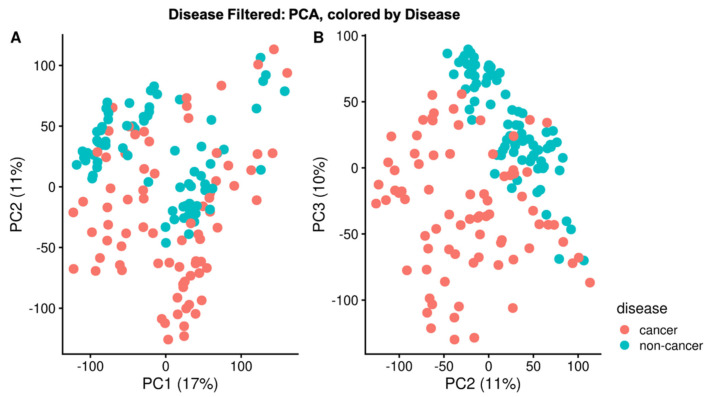
PCA by disease. PCA was performed on the normalized intensities of filtered corresponded peaks. Cancer and non-cancer samples separate partially along PC2 (Panel (**A**)) and more clearly along PC2 and PC3 (Panel (**B**)). This strongly suggests that PC2 and PC3 capture some of the biological variance between cancer and non-cancer Separation by disease class does not occur along PC1, instead, PC1 corresponds to the instrument on which the spectrum was acquired (Figure S1).

PCA performed using the normalized intensity of the corresponded peaks present in at least 25% of a sample class mostly separates cancer and non-cancer samples along principal components (PC) two and three (Figure 1A,B). Although imperfect, the presence of this decision boundary implies that these principal components reflect biological variance between disease classes. There is also a partial separation along PC1 that reflects if the spectrum was acquired on Fusion 1 or Fusion 2 (Supplemental Figure S1). This potential issue is mitigated by the fact that the cancer and non-cancer samples are very evenly split between instruments and the difference in variance between PC1 and PC2 is only 6%.

**Figure 2:**
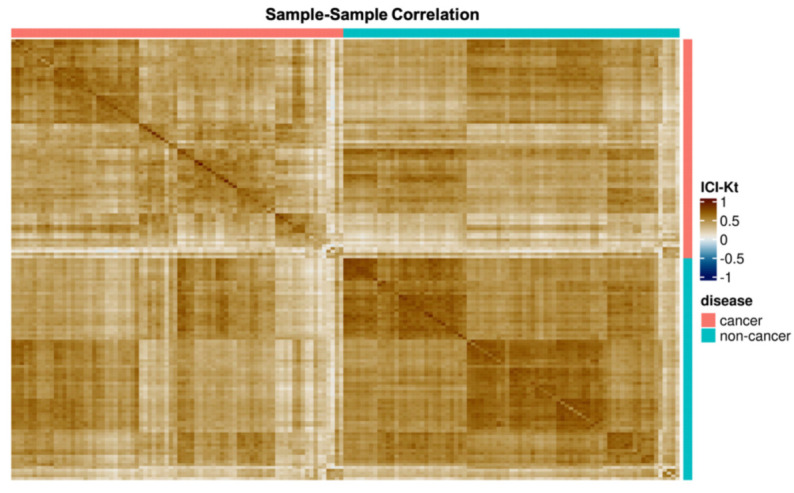
Correlation heatmap by disease. In general, samples belonging to the same disease class correlate more strongly with other samples in the same disease class. Stronger correlation patterns are observed within the non-cancer samples as compared to the cancer samples. In both cancer and non-cancer samples, there are multiple subgroups of samples that correlate more strongly with one another as well as occasional samples that have poor correlation with any other sample.

The observed correlation patterns among samples further support the presence of significant biological variance (Figure 2). Non-cancer samples correlate strongly with one another and are well differentiated from the cancer samples. Although cancer samples do cluster together, correlation within the cancer samples was weaker as compared to the non-cancer samples. Further investigation of the correlation among the cancer samples reveals two groups of cancer samples.

### 2.3. Differential Abundance of Lipid Categories between Cancer and Non-Cancer Lung Tissue

**Figure 3:**
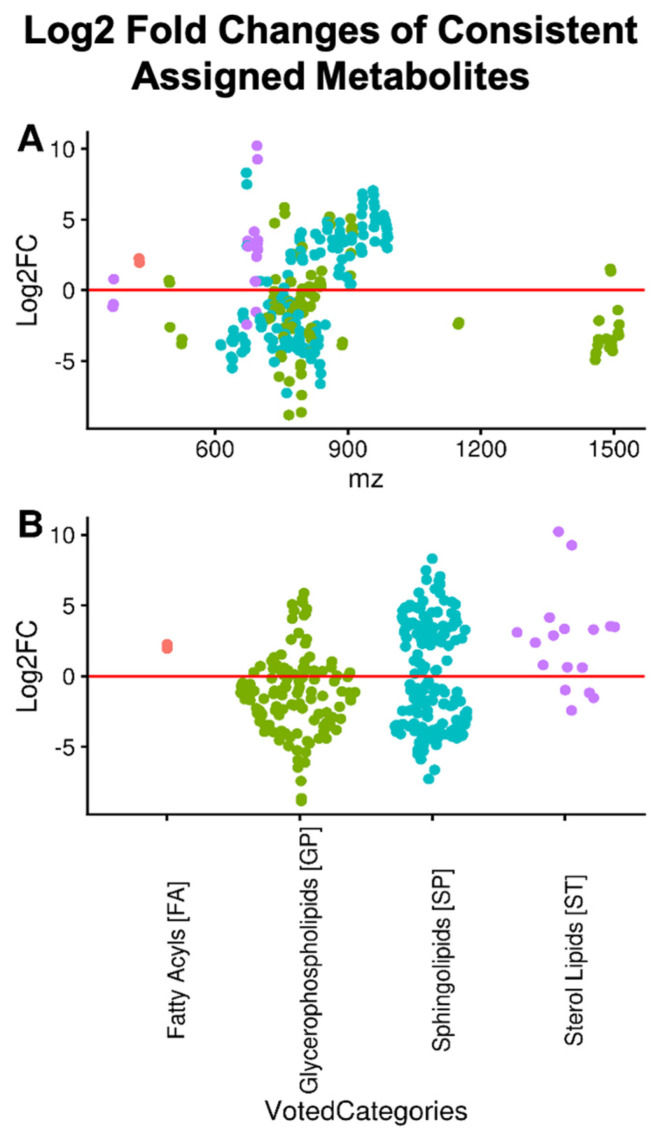
Log2 fold change by category and m/z. The Log2 fold-change (Log2FC) of corresponded peaks assigned to one lipid category are shown in panel (**A**) with respect to m/z and by class in panel (**B**). The extremely high fold-changes observed for some members of the sphingolipid and sterol lipid categories are due, in part, to imputed values. Most of the differentially abundant lipids occur in the 600 to 1000 m/z range; however, this region also has the highest density of assignments. Although no lipid category is exclusively more abundant, sterols are predominantly more abundant while substantial numbers of both sphingolipids and glycerophospholipids are more and less abundant.

Lipids from five lipid categories were observed: Fatty Acyls [FA], Glycerophospholipids [GP], Prenol Lipids [PR], Sphingolipids [SP], and Sterols [ST] across all samples. In addition, we created two new categories for Sphingolipids based on m/z low m/z (<700) and high m/z (>=700, see Figure 3), for a total of seven categories. Of these seven categories, only fatty acyls and prenols had no differentially abundant assignments. Additionally, few assignments were made to the fatty acyl or prenol categories. This may reflect the abundance of these lipids in our samples or their poor ionization in positive mode. The sterols and high m/z sphingolipids were significantly more abundant at the category level, whereas glycerophospholipids were significantly less abundant at the category level (Table 1).

Extremely high and extremely low log2 fold changes result from the imputation of missing values which occurs when an isotope-resolved molecular formula (IMF) is observed in one sample class but not the other. The relationship between log2 fold changes and m/z is shown in Figure 3. In the case of sphingolipids, more and less abundant sub-populations appear to correlate with m/z, with cancer having higher concentrations of higher m/z sphingolipids on average. Most of the sphingolipids past 700 m/z are of the phosphosphingolipid [SP03], a class that includes sphingomyelins, while before 700 m/z there is a mixture of both phosphosphingolipids [SP03] and ceramides [SP02]. Sterols and glycerophospholipids show no such pattern with respect to m/z. Querying the top 5 most abundant unique EMFs of the more abundant sterol IMFs against PubChem returned matches to known sterol esters. Additionally, 12 of the 13 sterol EMFs assigned in our study were classified into the ST01 class of sterols from LipidMaps, which includes sterol esters. The majority of the glycerophospholipids could not be classified uniquely into a single class; however, the majority of the glcyerophospholipids that were uniquely classified (19 of 32) were classified into the Glycerophosphoglycerophosphoglycerol [GP12] class that includes cardiolipin derivatives.

### 2.4. Lipid Category Correlation and Co-Occurrence Heatmaps

**Figure 4:**
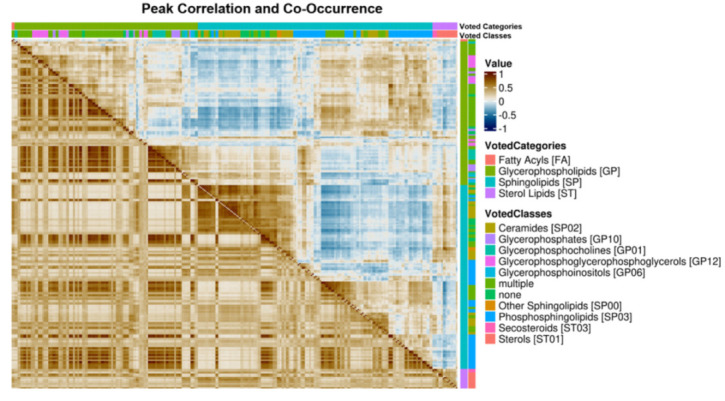
Peak correlation and peak co-occurrence combined heatmap. The upper-right corner shows the Kendall-tau correlation among consistently assigned lipid features. Strong intra-category correlation is observed for some lipids which possibly results from co-regulation. Shown in the bottom-left is the co-occurrence of consistently assigned lipid features. There are two sub-populations of sterols: one that co-occurs with a sub-population of sphingolipids and another that co-occurs with a sub-population of glycerophospholipids. The biological relevance of this co-occurrence is unclear. Alternatively, this co-occurrence may simply be artifactual, if these lipids were generally of lower relative abundance.

Kendall-tau correlation values between features were calculated using samples with non-zero corresponded peak normalized intensities for both features (Figure 4). Multi-classified features were dropped prior to analysis. At the lipid category level, we see distinct groups of correlated lipids within each category and in general, intra-category correlation is stronger than inter-category correlation. This observation suggests possible regulation of these lipids at a category level and implies that the majority of the corresponded peaks are consistently assigned to the correct lipid category.

Of the three differentially abundant categories of lipids, the glycerophospholipids collectively show the strongest intra-category correlations and some subgroups of glycerophospholipids are correlated with subgroups of sphingolipids and sterols. Within the sphingolipid category, there are distinct sub-populations of sphingolipids with differing amounts of intra-group correlation. Interestingly, the groups of sphingolipids that correlate well with sterols do not correlate strongly with other groups of sphingolipids. Finally, the sterols show the weakest intra-category correlation of the three categories, but there are two distinct subgroups of sterols correlated with one another, suggesting possible coregulatory mechanisms between these groups of lipids.

**Figure 5:**
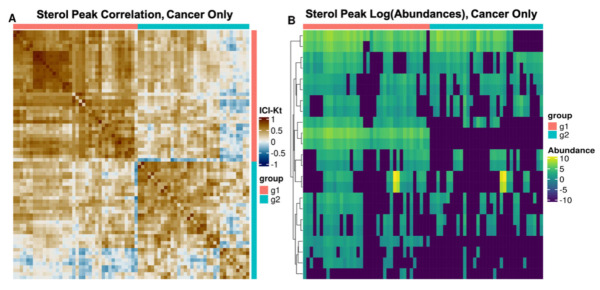
Sterol-only cancer sample-sample correlation heatmap. ICI-Kendall-tau correlation heatmap (**A**) and abundance heatmap across samples (**B**). Both plots show that the cancer samples can be separated into two groups based on the sample-sample correlation; however, these groups do not correspond to a histological subtype of NSCLC. As indicated in Figure 4, there also appear to be two main groups of sterol peaks in (**B**).

The lipid co-occurrence analysis revealed patterns that were not obvious from correlation alone. Mirroring the correlation analysis, there are two sets of co-occurring sphingolipids. The sterols co-occur with one sub-population of sphingolipids; however, it is not the sub-population of sphingolipids that were correlated with the sterols. By considering only the sterol features when calculating sample-sample correlations, two distinct subgroups of cancer samples with similar patterns of sterol abundances were observed (Figure 5).

Although prescription data was not available for all the patients in our study, a sizeable fraction of the study participants attested to taking statins (20 of 60) that could explain the lipid profile subgroups we observed. However, the composition of the patients with respect to statin-use between the two sterol subgroups, evaluated using a chi-squared test, was not significant (*p*-value = 0.952). Furthermore, there was no clear correlation between statin use and the abundances of consistently assigned lipid categories in either cancer or non-cancer samples (Supplemental Figure S2). Thus, we hypothesize that these patterns of correlation and co-occurrence between lipid category subgroups are not iatrogenic but instead suggest the presence of two or more groups of cancer samples with different lipid metabolic patterns and regulation.

## 3. Discussion

### 3.1. Sample Correlation Analysis Shows Evidence of Metabolic Reprogramming in NSCLC

Metabolic reprogramming is ubiquitous in cancer and the number of distinct pro-cancer metabolic dysregulations within a cohort of patients with the same type of cancer is likely vast. Thus, we hypothesize that the lipid profiles of cancer samples are expected to be less similar to one another than the lipid profiles of the non-cancer samples from the same patients. This hypothesis is analogous to previously observed high inter-tumor variance in gene expression [57], RNA editing [58], DNA methylation [59], and mitochondrial DNA content [60].

The correlation patterns observed in Figure 2 supports this hypothesis. Although some lipid profile differences may be attributed to patient genetic, metabolic or environmental variance, this would not account for the strong correlation patterns observed among samples of the non-cancer class. Correlation among the cancer samples was weaker as compared to non-cancer, consistent with our hypothesis, with several distinct clusters of correlation observed. Further analysis of these clusters did not show a correlation with NSCLC subtype. The limited amount of correlation observed between cancer and non-cancer samples is explained by the sizable effect of metabolic reprogramming on some components of cellular metabolism. Additionally, there were fewer common corresponded peaks in cancer than in non-cancer samples, suggesting more diverse lipid profiles among the cancer samples.

Furthermore, these correlation patterns demonstrate that our assignment methods and data analysis pipeline, including our quality control measures, are successfully reducing assignment ambiguity and providing consistent assignments across samples as well as instrument and clinical environment. Random incorrect molecular formula assignment or lipid classification would not produce the observed correlation patterns within disease classes or the stronger correlations observed among peaks classified to the same lipid category respectively.

### 3.2. Regulatory Interpretation of Lipid Category Correlation and Co-Occurrence

In addition to the differences in abundance at a lipid category level, our study also examined the correlation of normalized lipid intensity across samples as well as the co-occurrence of those features across samples. We observed a strong intra-category correlation among most glycerophospholipids, among several sub-populations of sphingolipids and weak intra-category correlation with the sterols but two distinct sub-populations. Additionally, we observed inter-category correlation among some glycerophospholipids, sphingolipids, and one sub-population of sterols.

After concluding that the observed lipid profile subgroups did not correspond to the use of statin medications, we hypothesize that these patterns are the result of co-regulation across lipid categories possibly in combination with regulation at a lipid category level. One possible candidate for this co-regulation is steroid response element-binding proteins (SREBPs) that regulate both sterol and glycerolipid biosynthetic pathways [61]. Both SREBP-1 and SREBP-2 contribute to the regulation of lipid biosynthesis [62] and SREBP-1 signaling due to B7-H3 overexpression has been correlated with increased glycerolipid production and aggressive NSCLC [63]. Weaker correlation patterns, such as those observed within sphingolipids and glycerophospholipids, could represent more complex regulatory mechanisms or a mixture of scavenging, de novo synthesis, and remodeling. Additionally, some correlation could be the result of multiple lipid categories sharing various precursor metabolites. Alternatively, these weaker correlation patterns could imply more incorrect assignments for those lipid categories, especially sphingolipids, which our lipid category classifier model tends to overpredict [55].

The co-occurrence analysis demonstrates that correlation paints an insightful but also incomplete picture. Many members of a given lipid category correlate strongly with other members belonging to the same category or class; however, not all members of the same class co-occur with one another. This is most evident within the sterols where there are two distinct co-occurring subpopulations of sterols (Figure 4) each of which also co-occurs with lipids of other categories effectively constituting two distinct sterol lipid profiles across the samples (Figure 5). These lipid profiles did not correspond to histological subtypes of NSCLC and their origin remains unclear.

### 3.3. Potential Clinical Implications

Our differential abundance analysis identified significant differences in the relative abundances of lipids between NSCLC tissue and non-cancerous tissue samples derived from the same patients. Notably, a subset of sterols (Figure 4) were significantly and consistently more abundant in the cancer samples compared to non-cancer. Sterol metabolism is unique among the various metabolic pathways implicated by these findings in that sterol metabolism is easily targetable with statins, a commonly prescribed class of pharmaceuticals. However, as discussed earlier, increased sterol production is observed across multiple cancers and as such, this finding is not unexpected. The mechanism by which statins confer a survival benefit in some NSCLC patients remains nebulous; however, increased sterol production is a prerequisite if statins exert this effect through inhibition of endogenous sterol production. Our results indirectly support this hypothesis; however, future studies are needed to test this hypothesis more conclusively. The data analysis pipeline used for this study could easily be adapted for the untargeted and comprehensive analysis of the effects of statins on lipid profiles.

Additionally, future studies are needed to investigate the mechanisms resulting in the lipid profile changes observed in our study. Substantial lipid profile changes involving multiple lipid categories could result from altered ACLY, an essential enzyme for general lipid biosynthesis [64], or SREBPs that regulate multiple lipid biosynthesis pathways [61,63] High sterol concentrations may result from mechanisms including EGFR activation (direct or indirect) which promotes sterol biosynthesis [65,66], while high sterol ester concentrations suggest SCD1 [67] or acetyl-CoA acetyltransferase (ACAT1) activity. If the presence of high sterol ester subtype of NSCLC can be confirmed, ACAT1 becomes a promising therapeutic target given that inhibition of ACAT1 has been shown to cause apoptosis in pancreatic cancer via a buildup of intracellular cholesterol and increased endoplasmic reticulum stress [68]. A similar mechanism may be useful in the treatment of NSCLC.

Lower cardiolipin concentrations may result from altered human cardiolipin synthase 1 [69], lysocardiolipin acyltransferase [70], or protein-tyrosine phosphatase mitochondrial 1 [71] expression. These enzymes are involved in cardiolipin biosynthesis and remodeling and have been observed to be differentially expressed in some NSCLC subtypes [72]. Altered cardiolipin metabolism may confer resistance to apoptosis in some subtypes of NSCLC given the key role of cardiolipins in apoptotic pathways [73]. The trend towards higher abundance high m/z phosphosphingolipids could indicate an increased production of specific sphingomyelins or decreased catabolism of those sphingomyelins. Given the various and opposing roles of sphingolipids in the development of cancer [74], further characterization of these differentially abundant lipids is necessary to understand their mechanistic role in NSCLC.

Therefore, multiple distinct mechanisms may result in the acquisition of a high concentration sterol, low glycerophospholipid, high m/z sphingolipid metabolic phenotype. This metabolic phenotype may be necessary for the development of NSCLC or simply a by-product of other disease processes. In the case of the former, pharmaceutical interventions targeting the metabolic phenotype directly may be useful for many genetically distinct NSCLC subtypes. Regardless, components of this metabolic phenotype may have utility as biomarkers for genetic subtypes of NSCLC.

## 4. Materials and Methods

### 4.1. Description of Paired Human NSCLC Cancer Samples and Mass Spectrometry Analysis

The collection, preparation, and mass spectrometry analysis of the paired cancer and non-cancer samples has been previously described (Sellers et al., 2015) [55]. In summary, cancer and non-cancer samples were obtained from eighty-six non-diabetic patients with suspected resectable stage I or IIA NSCLC. Written informed consent was collected from all subjects prior to inclusion and all samples were collected under a University of Louisville or University of Kentucky IRB protocol. Lipid extracts were prepared using a modified Folch extraction and reconstituted for direct infusion ultra-high resolution mass spectrometry on a pair of Thermo Tribrid Fusion Orbitrap instruments (FSN10115 and FSN10352, referred to as Fusion 1 and Fusion 2 respectively) coupled to an Advion nanoelectrospray system. 53 patients (102 spectra total) were acquired from Fusion 1 and 40 patients (77 spectra total) were acquired from Fusion 2. Fusion 1 samples were exclusively from the University of Louisville while Fusion 2 was a mix. Three of the patients had only a cancer or non-cancer sample acquired. De-identified patient and tumor demographics are described in Supplemental Table S1.

### 4.2. Molecular Formula Assignment and Lipid Characterization of Assigned Formulas

Our previously described SMIRFE algorithm [51] was used to assign molecular formulas to spectrally characterized peaks in an untargeted manner. The initial EMF database was generated using an m/z limit of 1605 m/z, and maximum numbers for each element were set to C: 130, N: 7, O: 28, P: 3, H: 230. Assigned formulas were allowed to have K, Na, H, and NH_4_ adducts (only positive mode samples were assigned). Assigned molecular formulas were then classified into one or more lipid categories using our lipid classifier tool [55]. Lipid classes that were previously observed to be overclassified by our models (neutral and acidic glycosphingolipids) were excluded.

### 4.3. Consistently Assigned Spectral Feature (Corresponded Peak) Generation and Peak Intensity Normalization

Our SMIRFE assignment method assigns isotope-resolved molecular formulas (IMFs) to characterized peaks in each spectrum. Each IMF represents an isotopologue of a given elemental molecular formula (EMF) (e.g., ^13^C_1_^12^C_5_^1^H_12_^16^O_6_ is an IMF representing the m+^13^C_1_ isotopologue of the EMF C_6_H_12_O_6_). Consistently assigned (i.e., corresponded peaks) are identified using an in-house method we have named EMF voting which is described in Appendix A. EMF voting identified 3485 total corresponded peaks across all 157 spectra with 855 corresponded peaks present in 25% or more of either the cancer or non-cancer group of samples.

To minimize false assignments and their effects, “not lipid” and multiply classified lipids were not considered in downstream analyses. All lipid isotopologue intensities were normalized by dividing the isotopologue intensity by the median intensity of all the peaks in the sample. For differential abundance analysis, missing values were replaced with a threshold value that is ½ of the lower confidence interval of the distribution of all log-transformed intensities from that tissue.

### 4.4. Quality Control of Patient Samples

Sample-sample correlations were calculated using an information-content-informed (ICI) Kendall-tau correlation that considers missing values in the calculation of concordance and discordance of pairs [75]. To determine possible outlier samples, each sample’s median ICI-Kendall-tau correlation to other samples with the same disease label, as well as the fraction of lipids that could be considered as a possible outlier were calculated for each sample [76]. Samples with low median correlation outlier values (lower than the lower limit minus the 1.5 times the interquartile range, as defined in the boxplot.stats R function) and high outlier lipid fraction (higher than the higher limit plus 1.5 times the interquartile range, as defined in the boxplot.stats R function) were removed from further analysis.

### 4.5. Differential Abundance Analysis

To minimize the effects of misassignment, only corresponded peaks are used in differential abundance analysis. Differential abundance analysis was performed using both Linear Models for Microarray Data (LIMMA) [77,78] and Semi-parametric Differential Abundance/Expression Analysis for Metabolomics (SDAMS) [79,80] on the normalized features (see above). No pairing of samples was considered for the differential analysis, given the large size of the dataset and the desire to detect differences that are distinct even across patient biological variance. For LIMMA, the linear model included both the disease status and instrument the sample was collected on, and then the contrast for disease status was extracted. This helps to control for the effect of the instrument, which is known to be larger than the effect of cancer status based on principal component analysis (see Figure 1 and Supplemental Figure S1). An adjusted p-value cutoff of 0.01 was used to determine metabolite significance. All lipid IMFs identified by either LIMMA or SDAMS were considered in this analysis. P-values were adjusted using the Benjamini–Hochberg multiple testing adjustment [81]. Cancer samples were clustered via hierarchical clustering on the sample-to-sample distances calculated as one minus the ICI-Kendall-tau correlation. Cluster leaves were ordered using the dendsort package [82]. All data manipulations and statistical testing were carried out in R v 4.1.0. [83].

LIMMA identified 356 differentially abundant corresponded peaks while SDAMS identified 481 abundant corresponded peaks. Together LIMMA and SDAMS identified 491 total unique differentially abundant corresponded peaks with 346 corresponded peaks identified by both methods. Of the 491 total unique differentially abundant corresponded peaks, 304 of them were assigned to one lipid category and the rest either had multiple lipid categories, were classified as “not lipid”, or a classification was not generated by the software. Only the 304 with a single lipid category were analyzed further. The log2 fold changes for each categorized corresponded peak between cancer and non-cancer was then calculated.

### 4.6. Lipid Category Enrichment of Statistically Significant Peaks

Using all of the peaks with a single assigned lipid category, hypergeometric enrichment of the significant peaks in each category was tested. This used the assigned lipid category as an annotation of the peak, and all of the singly categorized lipids as the peak universe (526 peaks). In addition to the lipid category, two more annotations were created for the sphingolipid [SP] category based on whether the lipid formula had an m/z less than or greater than/equal to 700 m/z. The statistically significantly more abundant and less abundant features (based on log2 fold-changes of cancer/non-cancer) were tested independently (more abundant, 131; less abundant, 173). Hypergeometric enrichment was performed using the categoryCompare2 R package (v 0.99.158) [84].

## 5. Conclusions

The combination of SMIRFE, EMF voting, and machine learning enable comprehensive lipid profiling in human-derived samples. The stronger within-patient correlation and within-lipid category and class correlations observed in our study confirm the accuracy of the molecular formula assignments and their predicted lipid classifications. Differential abundance analysis of the consistently assigned and classified lipid features identified a consistent metabolic profile difference between cancer samples and non-cancer controls. Cancer lipid profiles had consistently higher sterol and high-m/z sphingolipid relative concentrations and lower cardiolipin relative concentrations compared to neighboring non-cancer tissue. These profile differences can be partially explained by known examples of genetic lesions prevalent in NSCLC and common metabolic alterations observed in cancer metabolic reprogramming.

Furthermore, we hypothesize that the observed trend towards a high-sterol phenotype, combined with previous observations that statins and mevalonate pathway-altering drugs improve outcomes in some NSCLC patients, suggests that these drugs may be altering lipid profiles as part of their mechanism of action. Future experiments could test this hypothesis by utilizing our data analysis pipeline to detect and quantify the impact of pharmacological interventions on NSCLC lipid profiles. Additionally, genomic and transcriptomic studies on the same cohort of patients could identify genetic markers suggestive of this metabolic phenotype. In the case that multiple distinct genetic lesions converge to the same or similar metabolic phenotypes, therapies targeting the mevalonate pathway may have a role, either directly chemotherapeutic or adjuvant, in the treatment of many genetically distinct subtypes of NSCLC.

## Figures and Tables

**Table 1:** Differential abundance analysis results at the lipid category level.

Category	Total	More Abundant Features			Less Abundant Features		
		Expected	Observed	*p*-Adjust	Expected	Observed	*p*-Adjust
Fatty Acyls [FA]	12	2.989	2	1	3.947	0	1
Glycerophospholipids [GP]	205	51.055	37	1	67.424	88	**0.00503**
Prenol Lipids [PR]	5	1.245	0	1	1.644	0	1
Sphingolipids [SP]	281	69.983	79	0.09861	92.420	81	1
Sphingolipids [SP]–Low m/z	33	8.219	3	1	10.854	16	0.141
Sphingolipids [SP]–High m/z	248	61.764	76	**0.00967**	81.567	65	1
Sterol Lipids [ST]	23	5.728	13	**0.00643**	7.084	3	1

For each category of lipids, the number of observed more and less abundant features was recorded and compared to the number of expected more and less abundant features to statistically evaluate the differential abundance of that specific lipid category. The *p*-values were calculated using a hypergeometric test and adjusted for multiple testing using the Benjamini–Hochberg technique [56]. This revealed two statistically significant, more abundant lipid categories and one statistically significant, less abundant lipid category in cancer compared to non-cancer, which are bolded.

## Data Availability

All data and results presented here are available in a FigShare repository: DOI: https://www.doi.org/10.6084/m9.figshare.14199521.v3. This includes characterized peaks derived from Fourier transform mass spectra and all downstream analyses of these characterized peak lists.

## Supplementary Materials

The following are available online at https://www.mdpi.com/article/10.3390/metabo11110740/s1, Figure S1: PCA of Consistently Assigned Spectral Features by Instrument, Figure S2: Abundance heatmap of consistently assigned lipids in non-cancer and cancer samples, with statin use indicated, Table S1: Demographic Information NSCLC.

## Appendix A

EMF Voting description and IMF-level data extraction.

Elemental molecular formula (EMF) voting was used to match peaks across samples and determine the most likely assignments from SMIRFE. First, for each sample, the assignments are extracted, filtered, and scored. Those assignments containing sulfur are removed, and any assignments that contain only peaks that were marked as being questionable removed. Scores are calculated as 1–E-value, and EMFs that were classified as lipids had their score multiplied by 2.

For each sample, peaks were grouped to a sample specific EMF by determining the list of shared EMFs across a group of peaks (grouped_EMF). Scores for each formula in the grouped_EMF in the sample were taken as the best score for that formula from available scores in the group. After all sample grouped_EMFs are generated, additional scores for a formula are considered in actual voting by looking for the same unadducted base EMF with different adducts and adding these scores into the final total score.

The pseudo_EMFs are collections of grouped_EMFs across samples. They are generated across samples by iteratively merging grouped_EMFs with shared formulas, creating a new list of formulas in an EMF, and merging any pseudo_EMFs that have shared formulas again.

For each pseudo_EMFs, the most likely formulas are determined by voting. Voting uses the sum of EMF scores across samples, including those from the same formula with a different adduct. Those formulas with a total score in the top 90% of all total scores were considered “winning” formulas and kept as the “voted formula” set. Any peaks that did not originally have a “voted formula” were then checked to see if the peak location was within previously defined tolerance, and if the ordered peak intensities for the set of peaks from a sample are in the same order as the natural abundance probabilities of IMFs for the voted EMF. If so, then the peaks are kept as having the “voted formula” set.

After voting, all of the peaks are checked to make sure that the peak locations are within previously defined m/z specific cutoffs. Any peak outside of its specific cutoff is removed.

Finally, those pseudo_EMFs that share greater than 50% of their peaks are merged together, and voting on the EMFs is performed again.

Peak locations from our custom data processing pipeline are reported both in m/z and frequency, which are derived from the m/z values. The frequency values are more reliable (i.e., more consistent with less variance), but differ between instruments. Therefore, for each set of samples from a particular instrument, we use high confidence assignments (e-value <= 0.1, m/z <= 600) to derive a frequency cutoff using the mode of the distribution of frequency standard deviations across both groups of samples. This frequency cutoff is used to do EMF voting within an instrument. The m/z standard deviations of the voted peaks are then fit to m/z using a generalized additive model, and the standard deviation of the predicted m/z values are multiplied by 2 to derive an m/z cutoff so that voting can be performed across the instruments.

## References

[1] Kanitkar A.A., Schwartz A.G., George J., Soubani A.O. (2018). Causes of death in long-term survivors of non-small cell lung cancer: A regional Surveillance, Epidemiology, and End Results study. Ann. Thorac. Med..

[2] Molina J.R., Yang P., Cassivi S.D., Schild S.E., Adjei A.A. (2008). Non-small cell lung cancer: Epidemiology, risk factors, treatment, and survivorship. Mayo. Clin. Proc..

[3] Onoi K., Chihara Y., Uchino J., Shimamoto T., Morimoto Y., Iwasaku M., Kaneko Y., Yamada T., Takayama K. (2020). Immune checkpoint inhibitors for lung cancer treatment: A review. J. Clin. Med..

[4] Gettinger S., Choi J., Hastings K., Truini A., Datar I., Sowell R., Wurtz A., Dong W., Cai G., Melnick M.A. (2017). Impaired HLA class I antigen processing and presentation as a mechanism of acquired resistance to immune checkpoint inhibitors in lung cancer. Cancer Discov..

[5] Liu W.-j., Du Y., Wen R., Yang M., Xu J. (2020). Drug resistance to targeted therapeutic strategies in non-small cell lung cancer. Pharmacol. Ther..

[6] Walsh R.J., Soo R.A. (2020). Resistance to immune checkpoint inhibitors in non-small cell lung cancer: Biomarkers and therapeutic strategies. Ther. Adv. Med. Oncol..

[7] SEER*Explorer: An interactive website for SEER cancer statistics. https://seer.cancer.gov/explorer/..

[8] Uramoto H., Tanaka F. (2014). Recurrence after surgery in patients with NSCLC. Transl. Lung Cancer Res..

[9] Betticher D.C. (2005). Adjuvant and neoadjuvant chemotherapy in NSCLC: A paradigm shift. Lung Cancer.

[10] Pirker R., Krajnik G., Zöchbauer S., Malayeri R., Kneussl M., Huber H. (1995). Paclitaxel/cisplatin in advanced non-small-cell lung cancer (NSCLC). Ann. Oncol..

[11] Sandler A.B., Nemunaitis J., Denham C., Von Pawel J., Cormier Y., Gatzemeier U., Mattson K., Manegold C., Palmer M., Gregor A. (2000). Phase III trial of gemcitabine plus cisplatin versus cisplatin alone in patients with locally advanced or metastatic non–small-cell lung cancer. J. Clin. Oncol..

[12] Vansteenkiste J., De Ruysscher D., Eberhardt W., Lim E., Senan S., Felip E., Peters S., Group E.G.W. (2013). Early and locally advanced non-small-cell lung cancer (NSCLC): ESMO Clinical Practice Guidelines for diagnosis, treatment and follow-up. Ann. Oncol..

[13] Wozniak A.J., Crowley J.J., Balcerzak S.P., Weiss G.R., Spiridonidis C.H., Baker L.H., Albain K.S., Kelly K., Taylor S.A., Gandara D.R. (1998). Randomized trial comparing cisplatin with cisplatin plus vinorelbine in the treatment of advanced non-small-cell lung cancer: A Southwest Oncology Group study. J. Clin. Oncol..

[14] Paez J.G., Jänne P.A., Lee J.C., Tracy S., Greulich H., Gabriel S., Herman P., Kaye F.J., Lindeman N., Boggon T.J. (2004). EGFR mutations in lung cancer: Correlation with clinical response to gefitinib therapy. Science.

[15] Shepherd F., Pereira J., Ciuleanu T., Tan E., Hirsh V., Thongprasert S., Bezjak A., Tu D., Santabarbara P., Seymour L. (2004). A randomized placebo-controlled trial of erlotinib in patients with advanced non-small cell lung cancer (NSCLC) following failure of 1st line or 2nd line chemotherapy. A National Cancer Institute of Canada Clinical Trials Group (NCIC CTG) trial. J. Clin. Oncol..

[16] Chang A., Parikh P., Thongprasert S., Tan E.H., Perng R.-P., Ganzon D., Yang C.-H., Tsao C.-J., Watkins C., Botwood N. (2006). Gefitinib (IRESSA) in patients of Asian origin with refractory advanced non-small cell lung cancer: Subset analysis from the ISEL study. J. Thorac. Oncol..

[17] Crino L., Kim D., Riely G., Janne P., Blackhall F., Camidge D., Hirsh V., Mok T., Solomon B., Park K. (2011). Initial phase II results with crizotinib in advanced ALK-positive non-small cell lung cancer (NSCLC): PROFILE 1005. J. Clin. Oncol..

[18] Ferrara N., Hillan K.J., Novotny W. (2005). Bevacizumab (Avastin), a humanized anti-VEGF monoclonal antibody for cancer therapy. Biochem. Biophys. Res. Commun..

[19] Kim D.-W., Mehra R., Tan D.S.-W., Felip E., Chow L.Q.M., Camidge D.R., Vansteenkiste J.F., Sharma S., De Pas T., Riely G.J. (2014). Ceritinib in advanced anaplastic lymphoma kinase (ALK)-rearranged (ALK+) non-small cell lung cancer (NSCLC): Results of the ASCEND-1 trial. J. Clin. Oncol..

[20] Piperdi B., Merla A., Perez-Soler R. (2014). Targeting angiogenesis in squamous non-small cell lung cancer. Drugs.

[21] Sunshine J., Taube J.M. (2015). Pd-1/Pd-L1 Inhibitors. Curr. Opin. Pharmacol..

[22] Sgambato A., Casaluce F., C Sacco P., Palazzolo G., Maione P., Rossi A., Ciardiello F., Gridelli C. (2016). Anti PD-1 and PDL-1 immunotherapy in the treatment of advanced non-small cell lung cancer (NSCLC): A review on toxicity profile and its management. Curr. Drug Saf..

[23] Spigel D.R., Reckamp K.L., Rizvi N.A., Poddubskaya E., West H.J., Eberhardt W.E.E., Baas P., Antonia S.J., Pluzanski A., Vokes E.E. (2015). A phase III study (CheckMate 017) of nivolumab (NIVO; anti-programmed death-1 [PD-1]) vs docetaxel (DOC) in previously treated advanced or metastatic squamous (SQ) cell non-small cell lung cancer (NSCLC). J. Clin. Oncol..

[24] Hanahan D., Weinberg R.A. (2011). Hallmarks of cancer: The next generation. Cell.

[25] Hensley C.T., Faubert B., Yuan Q., Lev-Cohain N., Jin E., Kim J., Jiang L., Ko B., Skelton R., Loudat L. (2016). Metabolic heterogeneity in human lung tumors. Cell.

[26] Sellers K., Allen T.D., Bousamra M., Tan J., Méndez-Lucas A., Lin W., Bah N., Chernyavskaya Y., MacRae J.I., Higashi R.M. (2019). Metabolic reprogramming and Notch activity distinguish between non-small cell lung cancer subtypes. Br. J. Cancer.

[27] Sellers K., Fox M.P., Bousamra M., Slone S.P., Higashi R.M., Miller D.M., Wang Y., Yan J., Yuneva M.O., Deshpande R. (2015). Pyruvate carboxylase is critical for non–small-cell lung cancer proliferation. J. Clin. Investig..

[28] Faubert B., Li K.Y., Cai L., Hensley C.T., Kim J., Zacharias L.G., Yang C., Do Q.N., Doucette S., Burguete D. (2017). Lactate metabolism in human lung tumors. Cell.

[29] Hassanein M., Hoeksema M.D., Shiota M., Qian J., Harris B.K., Chen H., Clark J.E., Alborn W.E., Eisenberg R., Massion P.P. (2013). SLC1A5 mediates glutamine transport required for lung cancer cell growth and survival. Clin. Cancer Res..

[30] Metallo C.M., Gameiro P.A., Bell E.L., Mattaini K.R., Yang J., Hiller K., Jewell C.M., Johnson Z.R., Irvine D.J., Guarente L. (2012). Reductive glutamine metabolism by IDH1 mediates lipogenesis under hypoxia. Nature.

[31] Mohamed A., Deng X., Khuri F.R., Owonikoko T.K. (2014). Altered glutamine metabolism and therapeutic opportunities for lung cancer. Clin. Lung Cancer.

[32] Osugi J., Yamaura T., Muto S., Okabe N., Matsumura Y., Hoshino M., Higuchi M., Suzuki H., Gotoh M. (2015). Prognostic impact of the combination of glucose transporter 1 and ATP citrate lyase in node-negative patients with non-small lung cancer. Lung Cancer.

[33] Uramoto H., Osaki T., Inoue M., Taga S., Takenoyama M., Hanagiri T., Yoshino I., Nakanishi R., Ichiyoshi Y., Yasumoto K. (1999). Fas expression in non-small cell lung cancer: Its prognostic effect in completely resected stage III patients. Eur. J. Cancer.

[34] Huang J., Fan X.-X., He J., Pan H., Li R.-Z., Huang L., Jiang Z., Yao X.-J., Liu L., Leung E.L.-H. (2016). SCD1 is associated with tumor promotion, late stage and poor survival in lung adenocarcinoma. Oncotarget.

[35] Csanadi A., Kayser C., Donauer M., Gumpp V., Aumann K., Rawluk J., Prasse A., zur Hausen A., Wiesemann S., Werner M. (2015). Prognostic value of malic enzyme and ATP-citrate lyase in non-small cell lung cancer of the young and the elderly. PLoS ONE.

[36] Noto A., Raffa S., De Vitis C., Roscilli G., Malpicci D., Coluccia P., Di Napoli A., Ricci A., Giovagnoli M., Aurisicchio L. (2013). Stearoyl-CoA desaturase-1 is a key factor for lung cancer-initiating cells. Cell Death Dis..

[37] Visca P., Sebastiani V., Botti C., Diodoro M.G., Lasagni R.P., Romagnoli F., Brenna A., De Joannon B.C., Donnorso R.P., Lombardi G. (2004). Fatty acid synthase (FAS) is a marker of increased risk of recurrence in lung carcinoma. Anticancer Res..

[38] Wang Y., Zhang X., Tan W., Fu J., Zhang W. (2002). Significance of fatty acid synthase expression in non-small cell lung cancer. Zhonghua Zhong Liu Za Zhi Chin. J. Oncol..

[39] Sok M., Ravnik J., Ravnik M. (2009). Preoperative total serum cholesterol as a prognostic factor for survival in patients with resectable non-small-cell lung cancer. Wien. Klin. Wochenschr..

[40] Jamnagerwalla J., Howard L.E., Allott E.H., Vidal A.C., Moreira D.M., Castro-Santamaria R., Andriole G.L., Freeman M.R., Freedland S.J. (2018). Serum cholesterol and risk of high-grade prostate cancer: Results from the REDUCE study. Prostate Cancer Prostatic Dis..

[41] Kitahara C.M., de González A.B., Freedman N.D., Huxley R., Mok Y., Jee S.H., Samet J.M. (2011). Total cholesterol and cancer risk in a large prospective study in Korea. J. Clin. Oncol..

[42] Ringerike T., Blystad F.D., Levy F.O., Madshus I.H., Stang E. (2002). Cholesterol is important in control of EGF receptor kinase activity but EGF receptors are not concentrated in caveolae. J. Cell Sci..

[43] Hung M.-S., Chen I.C., Lee C.-P., Huang R.-J., Chen P.-C., Tsai Y.-H., Yang Y.-H. (2017). Statin improves survival in patients with EGFR-TKI lung cancer: A nationwide population-based study. PLoS ONE.

[44] Lin J.J., Ezer N., Sigel K., Mhango G., Wisnivesky J.P. (2016). The effect of statins on survival in patients with stage IV lung cancer. Lung Cancer.

[45] Han J.-Y., Lee S.-H., Yoo N.J., Hyung L.S., Moon Y.J., Yun T., Kim H.T., Lee J.S. (2011). A randomized phase II study of gefitinib plus simvastatin versus gefitinib alone in previously treated patients with advanced non–small cell lung cancer. Clin. Cancer Res..

[46] Diomede L., Albani D., Sottocorno M., Donati M.B., Bianchi M., Fruscella P., Salmona M. (2001). In vivo anti-inflammatory effect of statins is mediated by nonsterol mevalonate products. Arterioscler. Thromb. Vasc. Biol..

[47] Sadeghi M.M., Tiglio A., Sadigh K., O’Donnell L., Collinge M., Pardi R., Bender J.R. (2001). Inhibition of Interferon-Γ–Mediated Microvascular Endothelial Cell Major Histocompatibility Complex Class Ii Gene Activation by Hmg-Coa Reductase Inhibitors1. Transplantation.

[48] Weis M., Heeschen C., Glassford A.J., Cooke J.P. (2002). Statins have biphasic effects on angiogenesis. Circulation.

[49] Tsoumpra M.K., Muniz J.R., Barnett B.L., Kwaasi A.A., Pilka E.S., Kavanagh K.L., Evdokimov A., Walter R.L., Von Delft F., Ebetino F.H. (2015). The inhibition of human farnesyl pyrophosphate synthase by nitrogen-containing bisphosphonates. Elucidating the role of active site threonine 201 and tyrosine 204 residues using enzyme mutants. Bone.

[50] Mitchell J.M., Flight R.M., Wang Q.J., Higashi R.M., Fan T.W.-M., Lane A.N., Moseley H.N. (2018). New methods to identify high peak density artifacts in Fourier transform mass spectra and to mitigate their effects on high-throughput metabolomic data analysis. Metabolomics.

[51] Mitchell J.M., Flight R.M., Moseley H.N. (2019). Small Molecule Isotope Resolved Formula Enumeration: A Methodology for Assigning Isotopologues and Metabolite Formulas in Fourier Transform Mass Spectra. Anal. Chem..

[52] Moseley H.N., Carreer W.J., Mitchell J., Flight R.M. (2020). Method and system for identification of metabolites using mass spectra. U.S. Patent.

[53] Mitchell J.M., Fan T.W.M., Lane A.N., Moseley H.N.B. (2014). Development and in silico evaluation of large-scale metabolite identification methods using functional group detection for metabolomics. Front. Genet..

[54] Schrimpe-Rutledge A.C., Codreanu S.G., Sherrod S.D., McLean J.A. (2016). Untargeted metabolomics strategies—Challenges and Emerging Directions. J. Am. Soc. Mass Spectrom..

[55] Mitchell J.M., Flight R.M., Moseley H.N. (2020). Deriving Lipid Classification Based on Molecular Formulas. Metabolites.

[56] Benjamini Y., Hochberg Y. (1995). Controlling the false discovery rate: A practical and powerful approach to multiple testing. J. R. Stat. Soc. Ser. B (Methodol.).

[57] Hu Z., Fan C., Oh D.S., Marron J., He X., Qaqish B.F., Livasy C., Carey L.A., Reynolds E., Dressler L. (2006). The molecular portraits of breast tumors are conserved across microarray platforms. BMC Genom..

[58] Paz-Yaacov N., Bazak L., Buchumenski I., Porath H.T., Danan-Gotthold M., Knisbacher B.A., Eisenberg E., Levanon E.Y. (2015). Elevated RNA Editing Activity Is a Major Contributor to Transcriptomic Diversity in Tumors. Cell Rep..

[59] Hansen K.D., Timp W., Bravo H.C., Sabunciyan S., Langmead B., McDonald O.G., Wen B., Wu H., Liu Y., Diep D. (2011). Increased methylation variation in epigenetic domains across cancer types. Nat. Genet..

[60] Mizumachi T., Muskhelishvili L., Naito A., Furusawa J., Fan C.Y., Siegel E.R., Kadlubar F.F., Kumar U., Higuchi M. (2008). Increased distributional variance of mitochondrial DNA content associated with prostate cancer cells as compared with normal prostate cells. Prostate.

[61] Ericsson J., Jackson S.M., Kim J.B., Spiegelman B.M., Edwards P.A. (1997). Identification of glycerol-3-phosphate acyltransferase as an adipocyte determination and differentiation factor 1-and sterol regulatory element-binding protein-responsive gene. J. Biol. Chem..

[62] Wen Y.-A., Xiong X., Zaytseva Y.Y., Napier D.L., Vallee E., Li A.T., Wang C., Weiss H.L., Evers B.M., Gao T. (2018). Downregulation of SREBP inhibits tumor growth and initiation by altering cellular metabolism in colon cancer. Cell Death Dis..

[63] Luo D., Xiao H., Dong J., Li Y., Feng G., Cui M., Fan S. (2017). B7-H3 regulates lipid metabolism of lung cancer through SREBP1-mediated expression of FASN. Biochem. Biophys. Res. Commun..

[64] Zaidi N., Royaux I., Swinnen J.V., Smans K. (2012). ATP citrate lyase knockdown induces growth arrest and apoptosis through different cell-and environment-dependent mechanisms. Mol. Cancer Ther..

[65] Gabitova L., Gorin A., Astsaturov I. (2013). Molecular pathways: Sterols and receptor signaling in cancer. Clin. Cancer Res. Off. J. Am. Assoc. Cancer Res..

[66] Sukhanova A., Gorin A., Serebriiskii I.G., Gabitova L., Zheng H., Restifo D., Egleston B.L., Cunningham D., Bagnyukova T., Liu H. (2013). Targeting C4-demethylating genes in the cholesterol pathway sensitizes cancer cells to EGF receptor inhibitors via increased EGF receptor degradation. Cancer Discov..

[67] Bené H., Lasky D., Ntambi J.M. (2001). Cloning and characterization of the human stearoyl-CoA desaturase gene promoter: Transcriptional activation by sterol regulatory element binding protein and repression by polyunsaturated fatty acids and cholesterol. Biochem. Biophys. Res. Commun..

[68] Li J., Gu D., Lee S.S., Song B., Bandyopadhyay S., Chen S., Konieczny S.F., Ratliff T.L., Liu X., Xie J. (2016). Abrogating cholesterol esterification suppresses growth and metastasis of pancreatic cancer. Oncogene.

[69] Feng H.M., Zhao Y., Zhang J.P., Zhang J.H., Jiang P., Li B., Wang C. (2018). Expression and potential mechanism of metabolism-related genes and CRLS1 in non-small cell lung cancer. Oncol. Lett..

[70] Huang L.S., Kotha S.R., Avasarala S., VanScoyk M., Winn R.A., Pennathur A., Yashaswini P.S., Bandela M., Salgia R., Tyurina Y.Y. (2020). Lysocardiolipin acyltransferase regulates NSCLC cell proliferation and migration by modulating mitochondrial dynamics. J. Biol. Chem..

[71] Bao M.H.-R., Yang C., Tse A.P.-W., Wei L., Lee D., Zhang M.S., Goh C.C., Chiu D.K.-C., Yuen V.W.-H., Law C.-T. (2021). Genome-wide CRISPR-Cas9 knockout library screening identified PTPMT1 in cardiolipin synthesis is crucial to survival in hypoxia in liver cancer. Cell Rep..

[72] Sheng J., Zhao Q., Zhao J., Zhang W., Sun Y., Qin P., Lv Y., Bai L., Yang Q., Chen L. (2018). SRSF1 modulates PTPMT1 alternative splicing to regulate lung cancer cell radioresistance. EBioMedicine.

[73] Dudek J. (2017). Role of cardiolipin in mitochondrial signaling pathways. Front. Cell Dev. Biol..

[74] Ponnusamy S., Meyers-Needham M., Senkal C.E., Saddoughi S.A., Sentelle D., Selvam S.P., Salas A., Ogretmen B. (2010). Sphingolipids and cancer: Ceramide and sphingosine-1-phosphate in the regulation of cell death and drug resistance. Future Oncol..

[75] Flight R.M., Moseley H.N.B. ICIKendallTau. https://github.com/MoseleyBioinformaticsLab/ICIKendallTau.

[76] Gierliński M., Cole C., Schofield P., Schurch N.J., Sherstnev A., Singh V., Wrobel N., Gharbi K., Simpson G., Owen-Hughes T. (2015). Statistical models for RNA-seq data derived from a two-condition 48-replicate experiment. Bioinformatics.

[77] Phipson B., Lee S., Majewski I.J., Alexander W.S., Smyth G.K. (2016). Robust hyperparameter estimation protects against hypervariable genes and improves power to detect differential expression. Ann. Appl. Stat..

[78] Ritchie M.E., Phipson B., Wu D., Hu Y., Law C.W., Shi W., Smyth G.K. (2015). limma powers differential expression analyses for RNA-sequencing and microarray studies. Nucleic Acids Res..

[79] Li Y., Fan T.W., Lane A.N., Kang W.-Y., Arnold S.M., Stromberg A.J., Wang C., Chen L. (2019). SDA: A semi-parametric differential abundance analysis method for metabolomics and proteomics data. BMC Bioinform..

[80] Li Y., Wang C., Chen L. SDAMS: Differential Abundant Analysis for Metabolomics, Proteomics and Single-Cell RNA Sequencing Data., R Package Version 1.10.0; 2020. https://bioconductor.org/packages/devel/bioc/manuals/SDAMS/man/SDAMS.pdf.

[81] Thissen D., Steinberg L., Kuang D. (2002). Quick and easy implementation of the Benjamini-Hochberg procedure for controlling the false positive rate in multiple comparisons. J. Educ. Behav. Stat..

[82] Sakai R., Winand R., Verbeiren T., Moere A.V., Aerts J. (2014). dendsort: Modular leaf ordering methods for dendrogram representations in R. F1000Research.

[83] Ihaka R., Gentleman R. (1996). R: A language for data analysis and graphics. J. Comput. Graph. Stat..

[84] Flight R.M., Harrison B.J., Mohammad F., Bunge M.B., Moon L.D., Petruska J.C., Rouchka E.C. (2014). categoryCompare, an analytical tool based on feature annotations. Front. Genet..

[85] Mitchell J.M. (2019). Computational Tools for the Untargeted Assignment of FT-MS Computational Tools for the Untargeted Assignment of FT-MS Metabolomics Datasets. Ph.D. Thesis.

