# Supplementary material for: Untargeted Lipidomics of Non-Small Cell Lung Carcinoma Demonstrates Differentially Abundant Lipid Classes in Cancer vs. Non-Cancer Tissue
Source: Metabolites. 2021 Oct 28;11(11):740. doi: 10.3390/metabo11110740 (PMC8622625; doi:10.3390/metabo11110740)
Supplement: Supplementary file 1 [file metabolites-11-00740-s001.zip › metabolites-1433208-supplementary/NSCLC_lipidomics_supporting_material_v25.pdf]

Supporting Material for:

Untargeted lipidomics of non-small cell lung carcinoma demonstrates differentially abundant lipid classes in cancer vs non-cancer tissue

Joshua M. Mitchell[1,2,3,5,6], Robert M. Flight[2,3,5,7], and Hunter N.B. Moseley[1,2,3,4,5,*]

1 Department of Molecular & Cellular Biochemistry, University of Kentucky, Lexington, KY 40536, USA
2 Markey Cancer Center, University of Kentucky, Lexington, KY 40536, USA
3 Resource Center for Stable Isotope Resolved Metabolomics, University of Kentucky, Lexington, KY 40536, USA
4 Department of Toxicology and Cancer Biology, University of Kentucky, Lexington, KY 40536, USA
5 Institute for Biomedical Informatics, University of Kentucky, Lexington, KY 40536, USA
6 jmmitchell@lanl.gov
7 robert.flight@uky.edu
* Correspondence: hunter.moseley@uky.edu; Tel.: 859-218-2964

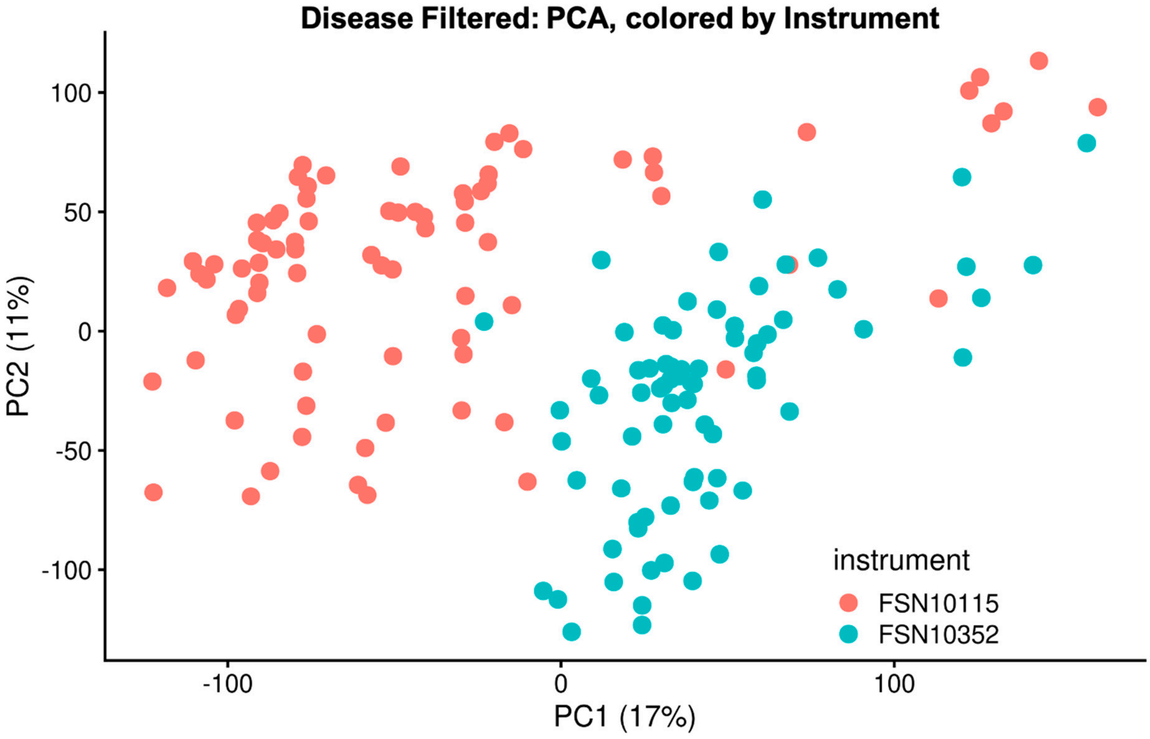

Figure S1. PCA of Consistently Assigned Spectral Features by Instrument. Principal component analysis (PCA) by instrument, which demonstrates a separation by instrument mostly along PC1, reflecting which instrument the spectra were acquired on.

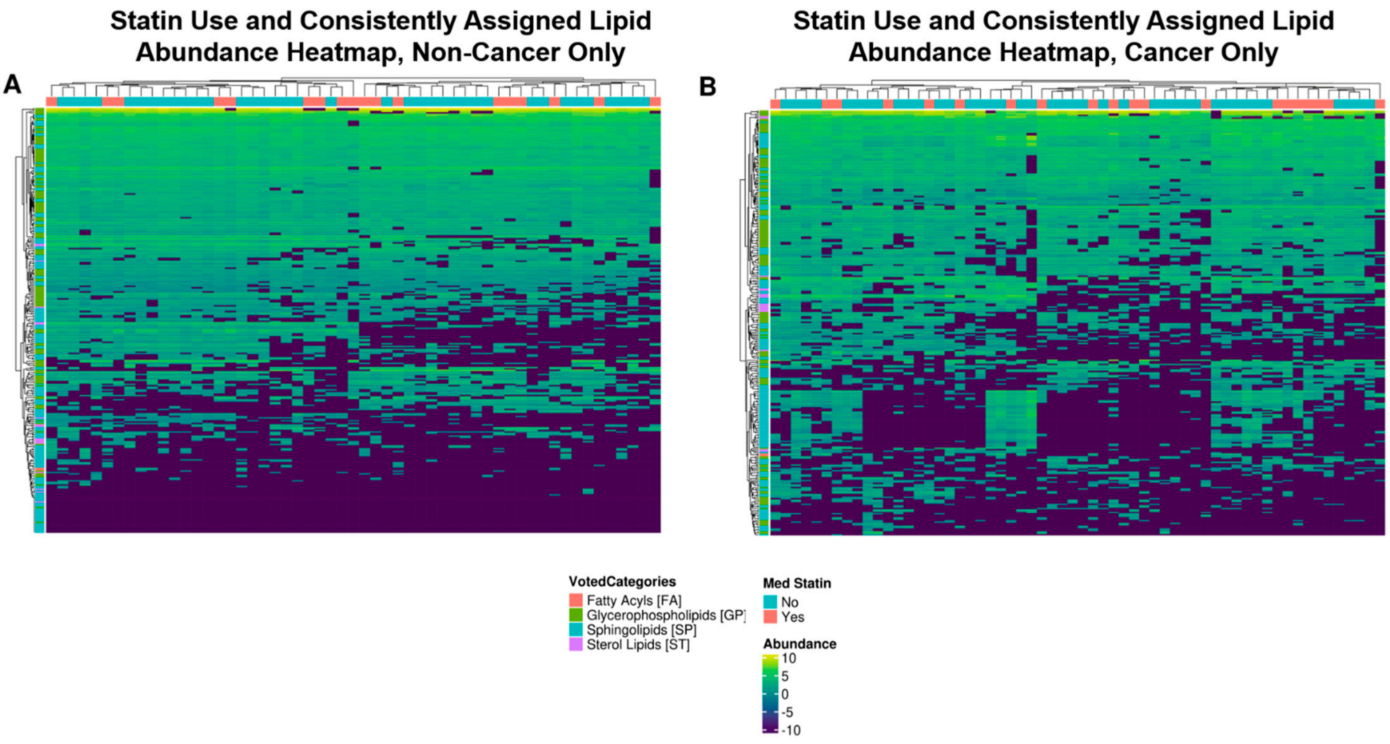

Figure S2. Abundance heatmap of consistently assigned lipids in non-cancer and cancer samples, with statin use indicated. A) Non-cancer samples. B) Cancer samples.
